# Peer-to–Patient-Aligned Care Team (Peer-to-PACT; P2P), a Peer-Led Home Visit Intervention Program for Targeting and Improving Long-term Care Services and Support for Veterans With High Needs and High Risk: Protocol for a Mixed Methods Feasibility Study

**DOI:** 10.2196/46156

**Published:** 2023-06-12

**Authors:** Sandra Garcia-Davis, Ana Palacio, Elizabeth Bast, Lauren S Penney, Erin Finley, Bruce Kinosian, Orna Intrator, Stuti Dang

**Affiliations:** 1 Department of Public Health Sciences University of Miami Coral Gables, FL United States; 2 Geriatric Research, Education, and Clinical Center (GRECC) Bruce W Carter Department of Veterans Affairs Medical Center Miami, FL United States; 3 Leonard M Miller School of Medicine University of Miami Miami, FL United States; 4 South Texas Veterans Health Care System San Antonio, TX United States; 5 Elizabeth Dole Center of Excellence for Veteran and Caregiver Research Washington, DC United States; 6 Greater Los Angeles Veterans Affairs Medical Center Los Angeles, CA United States; 7 Perelman School of Medicine University of Pennsylvania Philadelphia, PA United States; 8 Geriatrics and Extended Care Data Analysis Center (GECDAC) Canandaigua Veterans Affairs Medical Center Canandaigua, NY United States

**Keywords:** older veterans with high needs and high risk, peer support specialists, unmet needs, home visit, patient engagement, home services, care coordination

## Abstract

**Background:**

Keeping older veterans with high needs and high risk (HNHR) who are at risk of long-term institutional care safely in their homes for as long as possible is a Department of Veterans Affairs priority. Older veterans with HNHR face disproportionate barriers and disparities to engaging in their care, including accessing care and services. Veterans with HNHR often have poor ability to maintain health owing to complicated unmet health and social needs. The use of peer support specialists (peers) is a promising approach to improving patient engagement and addressing unmet needs. The Peer-to–Patient-Aligned Care Team (Peer-to-PACT; P2P) intervention is a multicomponential home visit intervention designed to support older veterans with HNHR to age in place. Participants receive a peer-led home visit to identify unmet needs and home safety risks aligned with the age-friendly health system model; care coordination, health care system navigation, and linking to needed services and resources in collaboration with their PACT; and patient empowerment and coaching using Department of Veterans Affairs whole health principles.

**Objective:**

The primary aim of this study is to evaluate the preliminary effect of the P2P intervention on patient health care engagement. The second aim is to identify the number and types of needs and unmet needs as well as needs addressed using the P2P needs identification tool. The third aim is to evaluate the feasibility and acceptability of the P2P intervention delivered over 6 months.

**Methods:**

We will use a quantitative-qualitative convergent mixed methods approach to evaluate the P2P intervention outcomes. For our primary outcome, we will conduct an independent, 2-tailed, 2-sample *t* test to compare the means of the 6-month pre-post differences in the number of outpatient PACT encounters between the intervention and matched comparison groups. Qualitative data analysis will follow a structured rapid approach using deductive coding as well as the Consolidated Framework for Implementation Research.

**Results:**

Study enrollment began in July 2020 and was completed in March 2022. Our sample size consists of 114 veterans: 38 (33.3%) P2P intervention participants and 76 (66.7%) matched comparison group participants. Study findings are expected to be published in late 2023.

**Conclusions:**

Peers may help bridge the gap between PACT providers and veterans with HNHR by evaluating veterans’ needs outside of the clinic, summarizing identified unmet needs, and developing team-based solutions in partnership with the PACT. The home visit component of the intervention provides *eyes in the home* and may be a promising and innovative tool to improve patient engagement.

**International Registered Report Identifier (IRRID):**

DERR1-10.2196/46156

## Introduction

### Background

Keeping older veterans with high needs and high risk (HNHR) who are at risk of long-term institutional care (LTIC) safely in their homes for as long as possible is a Department of Veterans Affairs (VA) priority [1,2]. The VA Geriatric and Extended Care Data Analysis Center (GECDAC) uses predictive modeling to identify veterans with HNHR at the highest risk of LTIC, hospitalization, or death [3]. The VA Geriatric and Extended Care (GEC) office recommends that veterans with HNHR be enrolled in long-term services and supports programs, including home- and community-based services, for best outcomes. Veterans with HNHR have complex needs that include not only physical but also mental, functional, and social needs, specifically frailty, social isolation, mobility challenges, and self-care deficits [4-8].

Older veterans with HNHR face disproportionate disparities and barriers to engaging in their care, which includes accessing care and services. Veterans with HNHR who are sicker and frailer often miss appointments and fail to visit the clinic for their care [9]. Their ability to maintain health is further complicated by complex unmet medical and social needs. When these needs go unmet, patients are at higher risk of adverse outcomes such as hospitalization, LTIC, and death [10-16]. Other adverse events include falls, inadequate nutrition, depression, incontinence, discomfort, and decreased quality of life [17,18]. Attending to the unmet health needs of older veterans with HNHR and planning services and solutions centered around what older people need based on *what matters most* to them is becoming an urgent public health priority [19,20]. Innovative interventions are needed to identify unmet needs and bridge the gap between needs and services in partnership with patients and family caregivers [1].

A promising approach to address these gaps at the VA concerns the use of peer support specialists (peers) [21-24]. Peers are veterans hired by the VA to help other veterans, usually those with serious mental illnesses, to engage them in their treatment successfully [25]. Peers are required to be veterans and must complete peer certification training. Peer certification training is conducted by a single contractor hired by the VA mental health services office. The training is at no cost to peers. Expected competencies include recovery principles, peer support practices, cultural competence, whole health approach to services, and advocacy. Continued education is required of all peers, including a minimum of 15 hours of competency-related training every year [25]. Peers promote recovery by using their common bond of being veterans and sharing their own recovery stories, providing encouragement, instilling a sense of hope, and teaching coping, empowerment, and social skills to veterans [25]. Peers understand the social and historical experiences shaping the veterans and connect with veterans in a different manner than the rest of their health care team. Being veterans themselves, peers are in a unique position to connect with veterans and assist with navigating the VA. Patient-aligned care teams (PACTs) at the VA are comprehensive medical homes that use a multidisciplinary team-based approach to effectively provide primary care and manage chronic conditions [26,27]. Preliminary studies suggest that embedded peers in mental health and primary care PACTs encourage veterans to be more active in managing their health [21-24,28-30]. Peers serve as role models, community connectors, and health care navigators. The preliminary success of the original peer support programs in specialty mental health led to congressional support for the creation of a peers-in-the-PACT program as part of the Maintaining Internal Systems and Strengthening Integrated Outside Networks (MISSION) Act of 2018 (section 506) with the implementation of peers at 30 VA sites [31]. Peers are relatively new members of the VA primary care PACT teams, and sites are working out the roles for peers in primary care.

Building upon the expansion of peers into VA primary care, we developed an innovative model for a peer-led home visit intervention to identify unmet needs in older veterans with HNHR as a home extension and *eyes in the home* for primary care PACTs called *Peer-to-PACT* (P2P). The peers work with the PACT to link veterans with HNHR to needed services and resources to address identified unmet needs. This paper describes the study protocol for a feasibility study to assess the preliminary effect of the P2P intervention on patient engagement and the factors that could affect its implementation in the VA setting.

### Study Goals

This study has three aims: (1) test the preliminary effect of the P2P intervention on patient engagement in care, measured by PACT outpatient health care use; (2) describe the number and types of needs and unmet needs identified, as well as needs addressed, among P2P participants; and (3) evaluate the feasibility and acceptability of the P2P intervention. The goal of the P2P intervention is to improve access to geriatric care and home- and community-based support services for older veterans with HNHR by providing patient-driven, proactive, personalized, and team-based care to link veterans and their caregivers to needed services and supports. We hypothesize that the P2P intervention will result in improvements in patient engagement by identifying unmet needs and addressing them.

## Methods

### Conceptual Design

The theoretical foundation of the P2P integrated intervention is grounded in the Andersen Healthcare Utilization Framework [32]. The Anderson model illustrates 3 main factors that influence the use of health care services: *predisposing, enabling*, and *need factors*. It incorporates the concept of mutability of factors, meaning that a factor with a high degree of mutability can be changed. Factors with a high degree of mutability, such as enabling resources, may thus justify deployment of resources. Therefore, the focus of our design emphasizes a peer-based approach to screening and identifying unmet needs in the context of the VA age-friendly health system (AFHS) mobility, mind, medications, and matters most (4Ms) framework as well as the fifth geriatric M, multicomplexity (mobility, mind, medications, matters most, and multicomplexity are together referred to as the 5Ms of geriatrics) [20,33], and then providing care coordination to link veterans with HNHR with services and resources to address their unmet health and social needs.

During the protocol development phase, we conducted a literature review of peer support and community health worker interventions, as well as assessments of the needs and unmet needs of older adults to draft the initial P2P intervention protocol. The final protocol and its component implementation materials were developed after iterative adaptations incorporating feedback from peers, PACT providers, veterans, advisory panel members, and various stakeholders.

### P2P Interventionists

Two certified VA peer support specialists were hired and paid through the VA mental health services office through usual VA hiring practices. Hired peers completed the P2P training program in addition to the training required for peer certification [25]. The P2P training program consists of 16 hours of didactic sessions covering topics such as veterans with HNHR, social determinants of health, activities of daily living, and VA and community resources, as well as 24 hours of hands-on training covering practice home visits, use of P2P data collection tools, and computerized patient record system (CPRS) documentation (discussed in further detail in the P2P Implementation Materials section).

### Advisory Panel

We established a national project advisory panel to guide intervention design, implementation, and dissemination activities. This panel includes key stakeholders from the VA offices of GEC as well as Mental Health and Suicide Prevention, the GECDAC, and the national VA peers in the PACT implementation team from the VA Center for Integrated Healthcare. The panel also includes subject matter experts in peer work at the VA, veterans with HNHR, home-based primary care, mental health, cultural competence, and non-VA community health worker programs, as well as Miami VA Healthcare System primary care PACT members. A biannual panel meeting was held to provide updates, solicit feedback, and consult one-on-one for specific matters.

### Study Setting

This P2P intervention feasibility study was conducted at the Miami VA Medical Center in Miami, Florida, United States.

### Study Design and Sample Size

This study uses a mixed methods (qualitative-quantitative convergent) design involving a nonrandomized 2-arm trial comparing the 6-month P2P intervention group with a 1:2 matched comparison group.

As this study is a pilot intervention, sample sizes were determined by what was deemed feasible within the available time frame and resources rather than power calculations. We aimed to recruit 35 veterans with HNHR or referred veterans for participation in the P2P intervention.

### Eligibility Criteria

To be eligible for the P2P intervention, veterans had to be aged ≥60 years, receiving care at the Miami VA Healthcare System, living within a 50-mile radius of the Miami VA facility, and not be planning to relocate in the next year. Veterans were excluded if they were enrolled in the VA home-based primary care program or residing in a hospice or nursing home. We also enrolled veterans who were referred by Miami VA Healthcare System PACT providers and met the eligibility criteria but were not on the HNHR list.

### Recruitment and Enrollment

Participant recruitment for this study is complete. Participants were enrolled into the P2P intervention at the Miami VA Healthcare System by peers. The peers recruited participants using three strategies: (1) calling eligible veterans identified from the quarterly GECDAC HNHR list; (2) mailing opt-in recruitment letters to veterans on the HNHR list; and (3) referrals made by PACT primary care clinicians, nurses, or social workers. In recruitment calls with eligible veterans, the peers described the P2P intervention to the veterans and how they might help the veterans manage their health conditions. If the veterans were interested, the peers obtained verbal consent to participate.

### Matched Comparison Group

Veterans in the matched comparison group were identified from the HNHR list, similar to the method used to enroll veterans into the P2P intervention. Comparison group veterans did not receive any peer services before or during the study period as evidenced by CPRS chart reviews and use data provided by the GECDAC. Comparison group participants were matched with P2P intervention participants on age, care assessment need score [34], JEN Frailty Index [35], and Nosos score [36,37] from the HNHR list for the period during which the P2P participant was recruited. Veterans in the comparison group received care as usual.

### P2P Intervention Overview

#### General Schedule

The P2P intervention is multicomponential and designed to support older veterans with HNHR to age in place. Peers provide usual peer support services (eg, facilitate peer support groups, advocate for veterans, and act as role models for recovery) in addition to the P2P intervention. The P2P intervention focuses on the home visit as an innovative tool to identify the unmet health and social needs of veterans with HNHR and linking them to solutions using an integrated P2P model. A general schedule of the intervention is presented in Table 1.

The intervention includes three main components detailed in the following sections: (1) needs identification via a peer-led home visit; (2) care coordination, health care system navigation, and linking to resources in collaboration with the PACT; and (3) patient empowerment and coaching using VA whole health principles.

**Table 1:** Peer-to–Patient-Aligned Care Team (P2P) intervention schedule.

When and what			How
**Rolling recruitment and enrollment**			
	Patient identification	HNHR^a^ list and PACT^b^ referrals	
	Eligibility screening	Filtering of HNHR list for age, distance from Miami VA^c^ Healthcare System, enrollment in HBPC^d^, and hospice or nursing home residence	
	Recruitment	Telephone calls, letters, or referrals	
	Week 1: scheduling home visit	Peers schedule a home visit with veterans enrolled in the P2P intervention via telephone calls or remote video visits	
	Week 2: preparing for the home visit	Peers complete a chart review of problem list, consults, medication list, and past notes in the last 30 days	
	Week 3: home visit	Peers identify needs via home visits using the P2P needs identification tool; when a home visit is not possible, needs are identified via a remote video visit	
	Week 4: P2P care coordination	Care coordination to address unmet needs is discussed between the peers and PACT during team huddles, via telephone calls or Microsoft Teams messaging, or in person	
**Months 2-5**			
	Patient education	Peers and veterans meet once every 2 weeks via telephone calls, remote video visits, or in person to review relevant topics of the Passport-2-Wellness, an educational book encompassing 8 health-related topics; the educational component is flexible in terms of the needs of the veterans, and peers have flexibility on deciding when to cover topics during the follow-up months	
	Follow-up on care coordination	Peers follow up on the status of identified unmet needs (eg, resolved or not resolved) with both the veteran and PACT and assist with unmet needs; peers follow up on referrals and consults and complete follow-up notes in the CPRS^e^	
**Month 6**			
	Discharge	Peers assess whether further follow-up is needed to continue advocating for the veteran; peers complete a P2P discharge summary indicating reason for discharge (eg, veteran does not require any additional peer support, or veteran has stopped communication with peer, or voluntarily withdrawn) and summary of participation in the program	

^a^HNHR: high needs and high risk.

^b^PACT: patient-aligned care team.

^c^VA: Department of Veterans Affairs.

^d^HBPC: home-based primary care.

^e^CPRS: computerized patient record system.

#### Needs Identification via a Peer-Led Home Visit

In preparation for the home visit, peers first ask pre–home visit safety questions, complete an initial contact note, and schedule a home visit. Before the home visit, peers review the patient’s VA CPRS chart to ascertain medical problems, existing and previous VA services, recent VA visits, hospitalizations, emergency use, no-shows and appointment cancellations, mental health history, cognition problems, medication list, social concerns, upcoming appointments, pending or cancelled consults and orders, and telehealth use.

During the home visit, peers provide additional information about the P2P intervention and verbally administer the needs identification tool, which is a health and social needs questionnaire (described in full detail in the P2P Implementation Materials section). Through conversations with, and observation of, the veteran and caregiver or caregivers, if available, as well as inspection of their home environment, the peers help the veteran to identify the key unmet needs that affect their overall health and well-being. During the visit, peers ask the veteran for permission to see different parts of their house. Specifically, peers ask to see bathrooms, living rooms, kitchens, floors, and entry ways. Peers use a home safety checklist within the needs identification tool to look for potential fall hazards (eg, loose cords, clutter, and carpets) and potential home safety equipment needs (eg, bath rails and entry ramp). The home visit takes approximately 2 hours. During the home visit, peers also provide the veteran with the Passport-2-Wellness, an educational book encompassing 8 health-related topics, and give them an overview of its contents (described in full detail in the P2P Implementation Materials section).

#### Care Coordination, Health Care System Navigation, and Linking to Resources in Collaboration With the PACT

The peers document the identified needs and a narrative of their observations in a templated CPRS note and add primary care PACT team members as cosigners. Peers collaborate with the appropriate PACT team member (primary care physician, nurse case manager, social worker, or pharmacist) to coordinate the ordering of needed consults and services. Care coordination to address unmet needs is discussed during team huddles (limited during the COVID-19 pandemic), via telephone calls or Microsoft Teams messaging, or in person.

Examples of unmet needs and linking to resources in collaboration with the PACT may include reaching out to the social work department for transportation concerns, linkage to food banks and meals on wheels for food insecurity concerns, or homemaker and home health aide for self-care concerns; reaching out to pharmacy for medication concerns; or working with the PACT nurse case manager or physician to refer veterans with falls or safety concerns to the falls clinic or order physical therapy, home safety assessment, or prosthetics for needed home safety durable medical equipment.

Peers may increase veteran health care engagement by bridging veterans back to their primary care team. Peers help veterans to navigate the health care system (eg, schedule appointments, send reminders, and provide guidance through the health care system bureaucracy) and provide emotional and social support (eg, identify VA and local social resources programs such as food banks and tenant advocacy). They continue to communicate with the veterans on the progress of their care plan and follow up on the status of the identified unmet needs (eg, resolved or not resolved) and assist with unresolved unmet needs by following up on referrals, consults, and orders.

#### Patient Empowerment and Coaching Using VA Whole Health Principles

During the initial visit and follow-up phase, the peers provide health and behavior coaching to the veterans. They review relevant sections of the Passport-2-Wellness with veterans as they deem fit and provide needed counseling in the context of the 5Ms of geriatrics [20]. They use VA whole health principles to teach veterans to “take charge of my life and health,” self-manage their chronic conditions, and take part in problem-solving. Peers also help the veterans to complete a VA whole health personal health inventory to assist them with setting goals, describing *what matters most* to them about their health, and identifying methods for filling unmet needs in alignment with their needs and preferences. Through continued contact via telephone, in person, or via videoconferencing, peers empower patients with problem-solving skills to manage their health, including self-care, medication adherence, nutrition, physical activity, and emotional well-being, and cover the relevant materials in the Passport-2-Wellness. Contacts last approximately 30 minutes and are tailored to meet patient goals and needs, access to services, and care coordination.

### Peer Assessment

Peers work with the veterans to ensure that they receive the needed services to enable them to remain in the community and address care fragmentation across providers and services. The P2P intervention is designed to be 6 months in duration, including 1 home visit during month 1 and contacts once every 2 weeks thereafter. The peers assess whether further follow-up is needed, past the 6 months, to continue advocating for the veteran or whether the veteran could be discharged from the P2P intervention. Peers complete a P2P discharge summary indicating the reason for the discharge (eg, veteran does not require any additional peer support, or veteran has stopped communication with peer, or voluntarily withdrawn) and a summary of participation in the program.

### Remote Care

In keeping with VA’s preference for remote care to enhance access and efficiency—and safety during the COVID-19 pandemic—we, too, implemented remote video visits by peers. Veterans are asked about interest in, and willingness to receive, a remote video visit, access to needed technology (internet and camera phone or computer), and help with technology access or use as required. For remote home visits, peers use the same in-person home visit template to identify unmet needs. During the remote home visit, peers ask veterans, whenever possible and safe to do so, to walk them through different parts of their house. Communication with the PACT follows the same process as outlined previously. Peers are provided tablet devices to be able to *telepresent* the veteran to a PACT provider at the VA site as needed and when possible. Peers may also work with social workers to provide veterans with tablet devices for remote visits if they qualify.

### P2P Implementation Materials

#### Overview

The protocol development phase focused on planning the P2P intervention protocol and its component implementation materials, including (1) a needs identification tool and a CPRS template for documentation, (2) the Passport-2-Wellness education and resource book, and (3) a peer training curriculum. These have all been implemented and refined using an iterative series of modifications with feedback from the peers, PACT providers, and local and national stakeholders. Two certified peer support specialists were actively engaged partners in the design and trial phases, and the process was modified to stay within their scope of peer work (eg, advocate for veterans, act as liaison between staff and veterans, provide outreach and education and work with clinical teams [23]).

#### Needs Identification Tool

To effectively address the needs of veterans with HNHR, we developed a needs identification tool to identify and understand the needs and unmet needs of older adults and improve patient engagement through a peer-led home visit. Peers initially conducted home visits using a longer questionnaire of validated questions to identify needs. The needs identification tool was modified to reflect the peers’ personalized holistic style using conversational language. The peers gather baseline information and identify unmet needs through a series of questions, conversations, and observations in the following domains: mobility, mind, medications, matters most, multicomplexity, and social determinants (Textbox 1). These needs domains were selected based on the 5Ms of geriatrics and previous studies showing that common barriers to patient engagement in outpatient programs include physical symptoms and limitations, mental illness, care fragmentation, isolation and lack of social support, financial insecurity, and poor social and neighborhood conditions [38-41]. These needs were then documented in a study-specific CPRS templated note to harmonize data collection and inform and coordinate care with the PACT regarding the veterans’ needs.

**Needs domains.**

Mobility and function

- Mobility, past falls, frailty, gait, and balance
- Activities of daily living and instrumental activities of daily living
- Homebound status
- Current use of support services

Mind

- Memory
- Mood (depression and anxiety)
- Substance use

Medications

- Medication insecurity, refills, questions
- Medication adherence concerns

Matters most

- Meaningful health goals

Multicomplexity

- Chronic medical conditions
- Chronic disease–related health care needs
- Hearing or vision problems
- Health care use

Social determinants

- Social support
- Transportation
- Food insecurity
- Financial stability
- Housing insecurity, housing density, neighborhood safety
- Legal concerns
- Technology access and ability to use technology

Home safety concerns and needs

- Outdoor grab bar
- Walker
- Assistive devices
- Bathroom assistive devices
- Smoke detector
- Presence of clutter, rugs, cords

#### The Passport-2-Wellness

We designed the Passport-2-Wellness educational book with 8 topics that were identified as being most common for, and most relevant to, older veterans with HNHR in the context of the 5Ms of geriatrics (Textbox 2). This Passport-2-Wellness book, which also contains a *Telehealth Visits* section and a section entitled *Healthcare Tools and Notes*, is provided to enrolled veterans as a resource and used by the peers as an educational tool. Peers, in collaboration with the veteran, select relevant topics to review during the intervention.

**Passport-2-Wellness educational topics.**

Mobility and Safety

- Barriers to Healthcare
- Independent Living
- Home Safety Checklist
- Falls Prevention

Caregiver Support

- Caregiver Support Tips
- Caregiver Resources
- Disaster Planning

Whole Health

- Introduction to Whole Health
- Your Personal Health Inventory
- Self-care and Mind-Body Techniques

Age-Friendly Health System 4Ms

- What Is Most Important to You?
- Memory Tips
- Mood Tips
- Medication Tips

Multicomplexity

- Common Chronic Conditions: Diabetes and Hypertension
- What is Diabetes?
- What Are the Symptoms of Diabetes?
- How Can You Manage Your Diabetes?
- Monitoring and Testing Your Blood Sugar
- Managing your Blood Sugar
- What My HbA1c Means
- How to Use a Glucometer
- What is Hypertension

Nutrition and Physical Activity

- Food as Medicine
- What Foods to Eat
- What Foods to Avoid
- Benefits of Physical Activity
- Tips to Increase Physical Activity
- Where Should You Start?
- Chair Yoga
- Community Fitness Zones

Health Communication

- Doctor Visit Tips and Shared Decision-Making
- Advance Directives

Care Plan and SMART Goals

- Care Plan Worksheet
- SMART Goals and Progress Worksheets

Extra Information

- Problem-Solving
- VA Claims, Disability and Pension
- Women Veterans Health Care
- Veteran Decision Aid for Care at Home or in the Community
- Community Dental Clinics
- Types of Vascular Disease
- Understanding Cholesterol
- Chronic Kidney Disease
- Congestive Heart Failure
- How You Can Reduce Heart Disease Risk
- Chronic Obstructive Pulmonary Disease (COPD)
- Understanding Insomnia

#### Peer Training Curriculum

Two hired peers were already certified peer support specialists [25]. There is onboarding training required for national peers in the PACT implementation team at all VA sites for usual peer activities. National peer training incudes competencies in recovery principles, peer support practices, cultural competence, communication, recovery and personal wellness goals, and whole health approaches to services. A complete list of expected knowledge and skill areas can be found in the *Peer Support Specialists Training Manual* [25]. To supplement this training, we developed an educational curriculum for the peers specific to the P2P intervention. This curriculum was developed integrating feedback from PACT providers, peers, key stakeholders, and the home-based primary care team. Overall, the duration of P2P intervention training is 40 hours: 16 hours for educational components and 24 hours for P2P hands-on training. Peer training was provided by multidisciplinary PACT and home-based primary care team members, including geriatricians, social workers, and nurses. Peers also received hands-on training in the use of VA CPRS, conducting chart review, and conducting a remote video visit. Peers made 3 home visits with the home-based primary care team to learn the process for conducting safe home visits, using personal protective equipment (because this intervention started during the COVID-19 pandemic) and checking for home safety and unmet needs. Some of the training was via *Talent Management System*, the VA’s enterprise-wide education, leadership development, learning, and employee development system. Training components are shown in Table 2.

**Table 2:** Peer support specialist Peer-to–Patient-Aligned Care Team (P2P) training curriculum.

Component			Required training			Duration (hours)		Modality
**P2P didactic training**								
	**Veterans with high needs and high risk**							
		Identifying the needs and unmet needs of veterans with high needs and high risk			1		Remote presentation	
		Social determinants of health			2		TMS^a^ course	
	Safety	Personal protective equipment training			1		In-person meeting with local occupational health	
	Mobility and functional limitations	Falls, assistive devices, ADLs^b^, and IADLs^c^			1		Remote presentation	
	Mental health	Managing depression, SUD^d^, and stress			2		In-person meeting with local recovery coordinator and mental health peers for information on VA^e^ resources and hospital tour	
	**VA** **vehicle**							
		Courses for driving a government vehicle			1		TMS course	
		Defensive driving, driving fundamentals, credit card, and so on (varies by facility)			1		TMS course	
	Motivational interviewing	Understanding good communication practices			1		TMS course	
	Social work	Social work services: transportation and long-term support services, VA and community resources, and caregiver resources			2		In-person meeting with local social worker	
	Telehealth	Remote care technology and tablet device use, digital divide consult, and mock remote video visit			2		TMS course	
	Research	VA required training in human participant research and HIPAA^f^			2		TMS course	
**P2P hands-on training**								
	Home visit	HBPC^g^ training and shadowing on home visit safety			12 (3 home visits)		In-person home visits with HBPC team	
	P2P tools	Use of the Passport-2-Wellness, needs data–gathering tools, and mock telephone calls			8		P2P team in person and remotely	
	VA records	Use of CPRS^h^ and chart review training			4		In person with program coordinator	
**Recommended training**								
	**Whole health**							
				Introduction to whole health, health coaching, and goal setting: SMART^i^ goals	2		TMS course	
				“Taking charge of my life and health” facilitator training	25 (5 days)		Remote learning (Zoom; Zoom Video Communications, Inc) and class discussion, partner activities, and small groups	
	Diabetes mellitus overview			About type 2 diabetes, hyperglycemia, hypoglycemia, and complications	2		In-person meeting with local diabetes nurse practitioner	
	Nutrition			Healthy eating, portion sizes, if you drink alcohol, and food shopping tour	4		In-person meeting with local dietician and nurse practitioner	

^a^TMS: Talent Management System.

^b^ADL: activity of daily living.

^c^IADL: instrumental activity of daily living.

^d^SUD: substance use disorder.

^e^VA: Department of Veterans Affairs.

^f^HIPAA: Health Insurance Portability and Accountability Act.

^g^HBPC: home-based primary care.

^h^CPRS: computerized patient record system.

^i^SMART: strategic, measurable, attainable, results-oriented, and time-bound.

### Data Collection

#### Demographic and Clinical Measures

The study team collected demographic, clinical, and use data from the CPRS and GECDAC on age, sex, race and ethnicity, marital status, care assessment need score [34], JEN Frailty Index [35], Nosos score [36,37], service-connected eligibility, 2-year emergency room visits, urgent care visits and primary care visits, and diagnosed conditions and services used among both intervention and comparison group participants (Table 3).

**Table 3:** Data collection.

Outcome measures						Description of measures		Data source		Schedule		
										Baseline		6 months
**Demographic and clinical data**												
		Demographics^a^				Age, sex, race, ethnicity, marital status, and rurality		CPRS^b^ chart review and GECDAC^c^ data pull		✓		N/A^d^
		Clinical characteristics^a^				Dementia, comorbidities, Nosos, CAN^e^, and JFI^f^, service-connected eligibility, and 2-year emergency room and urgent care		GECDAC data pull		✓		N/A
		Frailty				Fatigue, resistance, ambulation, illness, and loss of weight		Morley 5-item FRAIL^g^ scale [42]		✓		N/A
		Homebound status				Difficulty leaving the house and frequency of veteran leaving the house		NHATS^h^ homebound status questionnaire [38]		✓		N/A
**Aim 1: to test the preliminary effect of the intervention on patient engagement through PACT^i^ health care use**												
	Patient engagement: Health care use and support services use^a^				Primary care PACT encounters, consults, and support services (eg, homemaker and home health aide)		CPRS chart review and GECDAC data pull		✓		✓	
**Aim 2: to identify the number and types of needs and unmet needs as well as needs addressed using the P2P^j^ needs identification tool**												
	**Needs identification**											
			Health needs			ADLs^k^, IADLs^l^, and help needed; mobility needs; hearing, vision, and memory problems; and medication refills and adherence		Katz ADL Index [43], Lawton IADL Scale [44], and study-specific questions		✓		N/A
			Social needs			Food insecurity, housing insecurity, transportation, social isolation and loneliness, legal concerns, and technology needs		Two-item food insecurity screen [45], VA homeless screener [46], PRAPARE^m^ survey [47], UCLA^n^ Loneliness Scale [48], P2P legal concerns project-specific 1-item question, and VA digital divide consult		✓		N/A
			Home safety needs			Home safety devices need (eg, grab bars and bathroom assistive devices) and safety concerns (eg, unsafe steps and dark or poor lighting)		P2P project-specific checklist		✓		N/A
**Aim 3: to evaluate the feasibility and acceptability of the P2P intervention**												
	**Process evaluation**											
			Feasibility and acceptability			Quantitative: number of unique veterans referred and enrolled, average number of visits per veteran, average total number of consults placed per veteran, and average total number of GEC^o^ services received		CPRS chart review and GECDAC data pull		✓		✓
			Facilitators, barriers, improvements, and satisfaction			Qualitative: study-specific questions and semistructured interviews of veterans, PACT providers, peers, and leaders		P2P study-specific questionnaires, semistructured interviews, and periodic reflections		✓		✓

^a^Data collected for both intervention and comparison groups.

^b^CPRS: computerized patient record system.

^c^GECDAC: Geriatric and Extended Care Data Analysis Center.

^d^N/A: not applicable.

^e^CAN: care assessment need.

^f^JFI: JEN Frailty Index.

^g^FRAIL: fatigue, resistance, ambulation, illnesses, and loss of weight.

^h^NHATS: National Health and Aging Trends Study.

^i^PACT: patient-aligned care team.

^j^P2P: peer-to–patient-aligned care team.

^k^ADL: activity of daily living.

^l^IADL: instrumental activity of daily living.

^m^PRAPARE: Protocol for Responding to & Assessing Patients’ Assets, Risks & Experiences.

^n^UCLA: University of California Los Angeles.

^o^GEC: Geriatric and Extended Care.

#### Primary and Secondary Outcome Measures

Our primary outcome was patient engagement, defined as the number of outpatient PACT encounters. We obtained these data from the GECDAC and from CPRS chart reviews for both intervention and comparison groups. Patient engagement is a broad concept that combines positive patient behavior, such as obtaining preventive care, with patient activation [49]. In this study, we measured veteran engagement with the VA health care system by measuring participation in outpatient care and services. Peers collected data on frailty [42], homebound status [38], health needs, needs related to social determinants of health, and home safety needs. Secondary descriptive outcomes among intervention participants included prevalence and types of needs and unmet needs, actions taken to assist with meeting needs, and use of health care services, specifically services in the home (Table 3).

#### Implementation Measures

Quantitative process measures included number of unique veterans referred and enrolled, number of visits per veteran, total number of consults placed per veteran, and total number of GEC services received. We also conducted semistructured interviews at baseline and ongoing throughout the intervention period with key stakeholders (VA facility PACT providers and leadership, GEC leadership, GECDAC directors and staff, experts on VA peer support specialists, and national peers in the PACT implementation team). Detailed notes were taken on the interviews, and some of the interviews were audio recorded. The research team held ongoing periodic reflections [50] with the peers and study team to explore challenges and successful and unsuccessful strategies, as well as the peers’ perspective on their role as part of the clinical team [50]. In addition, study-specific questionnaires were used to collect data on satisfaction with the intervention as well as its feasibility and acceptability (Table 3).

### Data Analysis

We will use a quantitative-qualitative convergent mixed methods approach to evaluate P2P intervention outcomes. Specifically, we will use the reach, effectiveness, adoption, implementation, and maintenance (RE-AIM) framework to guide a formative process evaluation and assess the 5 key RE-AIM outcomes [51].

#### Quantitative Analysis

For our primary outcome, we will first calculate the difference in the number of primary care PACT outpatient encounters 6 months before and after enrollment for each participant in both intervention and comparison groups. An independent, 2-tailed, 2-sample *t* test will then be used to compare the means of the pre-post differences in the number of outpatient PACT encounters between intervention and comparison groups. The mean pre-post difference will be reported for each group with the corresponding SDs and 95% CIs. The folded F statistics will be evaluated to report either the pooled or Satterthwaite *P* value. We will calculate group-specific means and proportions for sociodemographic and clinical factors. At baseline, categorical variables will be compared between groups with the Pearson chi-square test or with the Fisher exact test for rare outcomes. The *t* test will be used to compare means across groups. *P* values will be reported, with lower values interpreted as more stable and less likely to be sporadic. All quantitative analyses will be performed using SAS 9.4 (SAS Institute Inc).

#### Qualitative Analysis

Qualitative data analysis will follow a structured rapid approach using deductive coding as well as the Consolidated Framework for Implementation Research (CFIR) [52]. A mixture of detailed note-taking and audio-recorded semistructured interviews will be used. Interviews will be transcribed and deidentified. Researchers will enter transcripts into NVivo 12 (QSR International), a software program used for qualitative and mixed methods research, and begin by reading each transcript repeatedly from beginning to end. To facilitate qualitative data coding and organization, we will use an NVivo project template, prepopulated with CFIR construct codes. Codes selected a priori include constructs within the following domains: outer setting, inner setting, and implementation process. Although constructs are selected a priori, we will allow for the expansion of codes and emergent themes as necessary.

To enhance validity and reliability of the qualitative data analysis, we will conduct exercise debriefings within the study team to evaluate potential differences in coding across analysts and resolve coding differences as needed. Running documents of all coding decisions, processes, and analyses will be kept, ensuring proper data management and coding practices.

### Ethics Approval, Participation, and Privacy Considerations

This study was approved by the Miami VA Healthcare System institutional review board (#1592752). No compensation was provided to veterans for participation. A waiver of Health Insurance Portability and Accountability Act (HIPAA) authorization was granted based on the determination that the risk to the privacy of individuals is minimal. To ensure privacy, efforts were taken to protect the identity of participants and ensure that data were kept confidential. Names, addresses, and social security numbers were replaced with a unique code and will only be maintained on a VA server; documentation of the procedure used to code the data will remain within the VA. All identifiers collected as part of this research project will be destroyed as per the records control schedule (RCS 10-1) of the Veterans Health Administration (VHA). Electronic files will be stored in folders with restricted access on a protected computer shared drive behind the VA firewall in a secure server. Nonelectronic records will be maintained in locked cabinets and behind locked doors. Information will be located in an area with limited public access. Data will not be transmitted as an attachment to unprotected email messages. The data will be accessible only to personnel involved in the study. All staff will be trained to avoid breach of confidentiality issues.

## Results

The P2P project was funded in October 2019. Study enrollment began in July 2020 and was completed in March 2022. Our sample size consists of 114 veterans: 38 (33.3%) P2P intervention participants and 76 (66.7%) matched comparison group participants. Data analysis is underway, and study findings are expected to be published in late 2023.

## Discussion

### Overview

Peers are increasingly being deployed in a variety of health care settings to provide patient-centered care. At the VA, efforts are underway to integrate peers in primary care settings. The role of peers is expanding and evolving, and peers are currently being proposed to be added in primary care PACTS at all VA facilities [53]. The use of peers in primary care settings at the VA is still relatively new, and innovative models for the use of peers are being developed at several VA sites based on local needs.

The P2P intervention aims to address unmet needs of older veterans with HNHR who are frail by addressing individual health and social needs as well as the home environment. Although peers are not medical providers, they are well suited to assess and understand patients’ perspectives and needs and are empowered to recommend patient-centric strategies to address them. This intervention aims to improve patient health care engagement through the timely use of needed services and supports. The protocol presented has undergone multiple modifications using feedback from peers, the PACT, stakeholders, and veterans. This study originally aimed to test the integrated P2P intervention using a structured 6-month experimental design. However, veterans with HNHR are a population with complex needs and with multimorbidities and poor health care engagement. They are challenging to treat because they often have more immediate health and living concerns that make participating in long interventions a last priority. Taking these challenges into consideration, a more flexible intervention schedule was developed in response to feedback from veterans with HNHR regarding their concerns with the intervention duration and time commitment required. In practice, the educational components are not constrained to specific weeks within the 6 months; instead, peers have flexibility on deciding when to cover topics within the allotted time frame. This flexibility takes into consideration the heterogeneity and complex needs of this population.

During the design phase, we used several strategies to improve recruitment: reducing the number of data collection instruments to reduce respondent burden, allowing peers to use their peer style to gather data while maintaining trust with the veteran, increasing provider knowledge of referrals to the P2P intervention, accepting patients referred by PACT providers, mailing recruitment letters, and giving peers flexibility to adapt the intervention to make it more veteran-centric as needed.

Peers are currently delivering the P2P intervention, conducting home visits, and identifying unmet needs as part of their routine clinical duties at the Miami VA Healthcare System. Because of the limitations with enrolling participants owing to COVID-19–related delays and the complexity of veterans with HNHR, we obtained permission to use clinical data from all veterans who participated in the P2P intervention. Our ability to track health care use and collect demographic and clinical data from electronic medical records presents us with the opportunity to assess the efficacy of the P2P intervention with data that are not easily collected via questionnaires. The results from this study will aid our understanding of the acceptability and feasibility of the P2P intervention and its effect on health-related outcomes.

### Strengths

The P2P intervention has several strengths. First, its novel approach of using peers in the PACT beyond the VA and into the home allows peers to serve as *eyes in the home* and identify needs that otherwise may continue to go unmet without proper care coordination. Second, using the AFHS and a whole health approach allows the peers and veterans to develop a personalized health plan based on individual values, needs, and goals. Third and last, prior VA research notes the need for functional and cognitive status as well as data related to social determinants of health to be a part of the routine electronic health record [54]. The needs identification tool we developed for this intervention was converted into a CPRS template with the goal of harmonizing data and coordinating care with the PACT on veterans’ unmet health and social needs.

### Limitations

As this is a nonrandomized study, the greatest challenge is the identification of an appropriate comparison group. We selected a matched comparison group from the same HNHR list from which the intervention participants were recruited to reduce selection bias. By selecting a matched comparison group that is similar according to a set of matching variables, we help to ensure that any observed differences in outcomes between the intervention and comparison groups can be more confidently attributed to the treatment itself, rather than other factors that may differ between the groups. The final sample size included 114 veterans: 38 (33.3%) P2P intervention participants and 76 (66.7%) matched comparison group participants. We made efforts to mitigate sample size limitations by including a 1:2 ratio of comparison group participants. In addition, the inclusion of referred patients may be deemed a deviation from the original protocol. However, the referred veterans will be assessed for comparable characteristics with veterans recruited from the HNHR list. Recent VA data demonstrate that using a combination of administrative data such as the HNHR list and clinician judgment is best suited for identifying patients for intensive care management interventions [54].

Safety precautions during the COVID-19 pandemic and home visits presented a potential risk for the intervention in terms of protection of the peers and the protection of older veterans with HNHR who are frail. The peers were trained by the home-based primary care team to make home visits and were trained on proper personal protective equipment use. Furthermore, peer use of remote care was limited by the access to, and willingness and ability to use, technology of the veteran with HNHR [55]. Peers used remote care where suitable. This aligns well with national VA priorities and stakeholder feedback.

### Conclusions

Peers may help bridge the gap between PACT providers and veterans with HNHR by evaluating veterans’ needs outside of the clinic, summarizing identified problems, and developing team-based solutions in partnership with the PACT. The home visit component of the intervention provides *eyes in the home* and may be a promising and innovative tool to identify unmet needs and home safety risks. The focus of our design emphasizes a community-based approach to successfully identify unmet needs and link individuals with HNHR with needed and effective health care services.

This project is timely because the *Veteran Peer Specialist Act of 2021* (Bill HR 4575) was recently approved by Congress to expand the peers-in-the-PACT program to all VA sites. The P2P intervention offers a protocol for integrating peers into the PACT that enhances and adds to the services provided by peers at the VA. The P2P intervention is being tested under real-world conditions using certified VA peer support specialists selected through usual VA hiring practices and assigned to the VA primary care service. The results collected from this study will provide important information on the effectiveness of a peer-led home visit intervention for veterans with HNHR who are considered vulnerable as an innovative strategy to identify need gaps using the AFHS 4Ms framework and coordinate care to increase timely access to needed services. Our findings will further gauge the feasibility of this framework in the existing health care system using peers. This intervention may highlight the innovative role that peers in the PACT may play to extend the reach of the PACT into the veterans’ homes to implement evidence-based long-term services and supports proactively and more equitably. Timely access to needed long-term care services and supports as a *preventive* measure may help meet veterans’ preference to age in place.

## Abbreviations

4Ms: mobility, mind, medications, and matters most
5Ms: mobility, mind, medications, matters most, and multicomplexity
AFHS: age-friendly health system
CFIR: Consolidated Framework for Implementation Research
CPRS: computerized patient record system
GEC: Geriatric and Extended Care
GECDAC: Geriatric and Extended Care Data Analysis Center
HIPAA: Health Insurance Portability and Accountability Act
HNHR: high needs and high risk
LTIC: long-term institutional care
MISSION: Maintaining Internal Systems and Strengthening Integrated Outside Networks
P2P: peer-to–patient-aligned care team
PACT: patient-aligned care team
RE-AIM: reach, effectiveness, adoption, implementation, and maintenance
VA: Department of Veterans Affairs
VHA: Veterans Health Administration

## Data Availability

The data sets generated and analyzed during this study are not publicly available owing to Department of Veterans Affairs and ethical redactions as well as redaction of ethically sensitive information; however, instructions on obtaining the data are available upon request.
